# KIT Mutations Are More Common in Mucosal than Acral Melanoma: A Case-Series of 152 Patients from a Single Centre

**DOI:** 10.3390/biomedicines14071434

**Published:** 2026-06-24

**Authors:** David Millán-Esteban, Ana Arauz-Gil, Zaida García-Casado, Víctor Traves, Eduardo Nagore, Celia Requena

**Affiliations:** 1 School of Medicine, Universidad Católica de Valencia San Vicente Mártir, C/Quevedo, 2, 46001 Valencia, Spain; 2 Department of Dermatology, Fundación Instituto Valenciano de Oncología, C/Profesor Beltrán Báguena, 8, 46009 Valencia, Spain; 3 Laboratory of Molecular Biology, Fundación Instituto Valenciano de Oncología, C/Profesor Beltrán Báguena, 8, 46009 Valencia, Spain; 4 Department of Pathology, Fundación Instituto Valenciano de Oncología, C/Profesor Beltrán Báguena, 8, 46009 Valencia, Spain

**Keywords:** melanoma, acral, mucosal, prevalence, KIT

## Abstract

**Background/Objectives:** Acral (AM) and mucosal melanomas (MM) have certain genetic similarities compared to non-acral cutaneous melanomas and share a restricted influence of UV radiation in their etiology. Yet, AM and MM arise in quite different locations from a histological perspective. This study aimed to report differences between AM and MM for the most prominently mutated genes in melanoma: *BRAF*, *NRAS*, *KIT*, and *TERT* promoter. **Methods:** We conducted a retrospective, single-center study including 152 patients diagnosed with AM (n = 121) or MM (n = 31) at the Fundación Instituto Valenciano de Oncología between 2000 and 2024. Clinical and histopathological data were collected, and mutational analyses of *BRAF*, *NRAS*, *KIT*, and the *TERT* promoter were performed using targeted sequencing. **Results:** MM presented with significantly greater Breslow thickness and higher rates of hematogenous metastasis compared to AM. While *BRAF*, *NRAS*, and *TERT* promoter mutations were similarly distributed between subtypes, *KIT* mutations were significantly more frequent in MM (33% vs. 12.3%; *p* = 0.043) and exhibited a broader mutational spectrum. Survival outcomes were poorer in MM, with lower 5- and 10-year survival rates compared to AM. **Discussion:** Despite shared UV-independent biology, AM and MM differ in clinically and molecularly meaningful ways. The higher prevalence and diversity of *KIT* mutations in MM suggest subtype-specific oncogenic mechanisms and potential therapeutic implications. These findings support a more refined classification of UV-independent melanomas based on anatomical and molecular distinctions.

## 1. Introduction

Melanoma is a highly aggressive malignancy originating from neural crest-derived melanocytes and accounts for a disproportionate share of skin cancer-related mortality worldwide [1]. While major advances in early detection and systemic therapies—particularly immune checkpoint inhibitors and targeted therapies against the MAPK pathway—have markedly improved outcomes for many patients, melanoma remains a complex disease [2]. Clinical behaviour, prognosis, and therapeutic response vary substantially across melanoma subtypes, reflecting differences in anatomical location, etiologic factors, and underlying molecular drivers [3].

From both clinical and molecular perspectives, melanoma is a complex biological entity with a spectrum of altered pathways. Cutaneous melanomas arising on intermittently sun-exposed skin typically display a high tumour mutational burden driven by ultraviolet (UV) radiation and are enriched for activating mutations in *BRAF* [4]. In contrast, melanomas developing in sun-protected sites—including acral skin and mucosal surfaces—exhibit markedly different genomic architectures, characterized by a low UV mutational signature, reduced point mutation burden, and a greater reliance on chromosomal instability and copy number alterations as oncogenic mechanisms [5].

Acral melanoma (AM) is a rare subtype of cutaneous melanoma that arises on glabrous, non-hair-bearing skin of the palms, soles, and nail apparatus. Although it represents only a small fraction of melanomas in Western populations, AM is the predominant melanoma subtype in individuals of African, Asian, and Hispanic ancestry [6]. Clinically, acral melanomas are often diagnosed at more advanced stages, with increased Breslow thickness and ulceration, contributing to poorer melanoma-specific survival compared with non-acral cutaneous melanoma. At the molecular level, AM is distinguished by a low tumour mutational burden and by recurrent structural genomic alterations affecting genes such as *KIT*, *CDK4*, *CCND1*, *TERT*, and *MDM2*; loss of tumour suppressor genes such as *CDKN2A* has also been reported, further highlighting the importance of copy number-driven oncogenesis in this melanoma subtype [7].

Mucosal melanoma (MM) represents an even rarer and more aggressive melanoma subtype, arising from melanocytes located within mucosal membranes of the head and neck, anorectal region, and genitourinary tract [8]. MM is frequently diagnosed at advanced stages due to its concealed anatomical location and the absence of well-defined precursor lesions, resulting in markedly worse survival outcomes compared with cutaneous melanoma [9]. Similar to acral melanoma, MM is not associated with UV exposure and demonstrates a low mutational burden, with recurrent alterations involving *KIT*, *NF1*, *CDKN2A*, and components of the PI3K–AKT pathway, as well as extensive chromosomal aberrations [10].

Because of these shared features—UV-independent etiology, low tumour mutational burden, and enrichment for structural genomic alterations—acral and mucosal melanomas are frequently discussed together as biologically related entities and are often grouped within the same molecular or therapeutic frameworks [11]. However, this conceptual proximity contrasts sharply with their profound histological and microanatomical differences. Acral melanomas arise in skin characterized by a thick and highly specialized stratum corneum, adapted to mechanical stress and friction, whereas mucosal melanomas develop in non-keratinized or variably keratinized epithelia that lack a true stratum corneum and are embedded in distinct immunological and stromal environments [5].

These differences in epithelial architecture, barrier function, and local microenvironment are likely to influence melanocyte biology, exposure to mechanical or inflammatory stimuli, and interactions with surrounding immune and stromal cells. Consequently, it remains unclear whether the commonly assumed biological similarity between acral and mucosal melanomas extends to their genetic underpinnings, or whether these histological disparities translate into meaningful differences in driver mutation profiles and tumor evolution.

In this context, the present study aims to directly compare the mutational status of key melanoma-associated genes—including *BRAF*, *NRAS*, *KIT*, and the *TERT* promoter—between acral and mucosal melanomas in a large, single-center cohort. By interrogating whether tumors arising in these distinct epithelial contexts share or diverge in their core genetic alterations, we seek to refine the molecular distinction between acral and mucosal melanoma and to contribute to a more biologically grounded classification of UV-independent melanoma subtypes.

## 2. Materials and Methods

### 2.1. Study Design and Patient Selection

This retrospective, single-centre observational study included patients diagnosed with acral melanoma or mucosal melanoma at the Department of Dermatology from the Fundación Instituto Valenciano de Oncología (Valencia, Spain) between January 2000 and December 2024. Inclusion criteria comprised histologically confirmed melanoma, classification as acral or mucosal melanoma based on anatomical location and histopathological features, and availability of clinical, histological, and molecular data. Only primary tumours from patients who had not received neo-adjuvant treatment were included. Cases with insufficient available tissue for molecular analysis or with incomplete clinical information were excluded.

Acral melanoma was defined as melanoma arising on glabrous skin, including palms, soles, nail apparatus, and interdigital areas, with histological features consistent with acral lentiginous melanoma. Mucosal melanoma was defined as melanoma arising from mucosal surfaces, including head and neck, anorectal, and genitourinary locations.

### 2.2. Clinical and Histopathological Data Collection

Clinical and demographic variables were retrieved from the database at the Department of Dermatology from FIVO, which collects information from routine clinical care. These included age at diagnosis, sex, primary tumour location, family history of cancer, presence of ulceration, microscopic satellitosis, lymphatic metastasis, and hematogenous metastasis. Histopathological variables were retrieved from pathology reports and included Breslow thickness, Clark level, and mitotic rate.

### 2.3. Molecular Analysis

DNA was extracted from three 10 µm-thick sections of formalin-fixed paraffin-embedded tumour samples obtained from primary lesions using QIAamp DNA FFPE Tissue Kit (QIAGEN, Hilden, Germany). DNA quality and integrity were assessed either by agarose gel electrophoresis, spectrophotometry, or automated electrophoretic systems. Molecular characterization focused on the mutational status of *BRAF*, *NRAS*, *KIT*, and the promoter region of *TERT*, using Sanger sequencing, or the Next Generation Sequencing Solid Tumor Solutions (Sophia Genetics™, Rolle, Switzerland) as described elsewhere [12]. Sequencing data was analyzed in-house using Sequencing Analysis Software 7 (Applied Biosystems™, Waltham, MA, USA) and Sophia DDM™ (Sophia Genetics™, Rolle, Switzerland) for Sanger and NGS, respectively. The variant allele frequency (VAF) threshold was 15% for Sanger and 5% for NGS.

### 2.4. Statistical Analysis

Statistical analyses were performed using IBM SPSS Statistics software (version 20.0; IBM Corp., Armonk, NY, USA). Descriptive statistics were used to summarize clinical, histopathological, and molecular characteristics. Categorical variables were expressed as frequencies and percentages, while continuous variables were summarized using medians and ranges.

Comparisons between acral and mucosal melanoma groups were performed using the chi-square test or Fisher’s exact test when appropriate. Statistical significance was defined as a two-sided *p*-value < 0.05. Survival analyses were conducted using the Kaplan–Meier method, and differences in survival between groups were evaluated using the log-rank test, considering death as the event for 5- and 10-year survival rates.

## 3. Results

A total of 152 patients diagnosed with acral melanoma or mucosal melanoma were included in the study, comprising 121 AM and 31 MM cases. The median age at diagnosis was 64 years for AM and 66 years for MM. Both subtypes showed a predominance of female patients, accounting for 52.9% (64/121) in AM and 67.7% (21/31) in MM.

The distribution of clinical and histopathological variables between AM and MM can be seen in Table 1. Briefly, a higher Breslow thickness (>2 mm) was significantly less common in AM than MM (50% vs. 85%; *p* = 0.004); though no statistically significant difference was found between AM and MM for ulceration (43.5% vs. 63.6%; *p* = 0.083), microscopic satellitosis (3.55 vs. 9.1%; *p* = 0.253), and lymphatic metastases (29.6% vs. 40.0%; *p* = 0.361). However, hematogenous metastasis was significantly less prevalent in AM than in MM (3.5% vs. 9.1%; *p* < 0.001).

Mutational analyses showed no statistically significant difference between AM and MM for *BRAF* (15.0% vs. 4.8%; *p* = 0.292), *NRAS* (14.3% vs. 14.3%; *p* = 1.00), and *TERT* promoter (3.2% vs. 12.5%; *p* = 0.185). Contrarily, *KIT* mutations were significantly less frequent in AM than in MM (12.3% vs. 33.0%; *p* = 0.043). *KIT* mutations in AM included dupl. 576–577, L576P, L642E, and V560D; while *KIT* mutations in MM included K642E, L576P, L642E, T488K, V559D, and Y646D.

The median survival rates at 5 and 10 years were 72.1% and 54.4% (CI 95%; 91.18–173.75) for AM, and 58.6% and 21.1% (CI 95%, 52.47–76.06) for MM, respectively.

## 4. Discussion

In this study, we present a large single-centre cohort of acral melanoma (AM) and mucosal melanoma (MM) patients with integrated clinical, histopathological, and molecular characterization, focusing on key melanoma-associated genes (BRAF, NRAS, KIT, and TERT promoter). Our findings suggest that, despite sharing several biological features—such as a low tumour mutational burden and a limited role of ultraviolet radiation—AM and MM differ in the relative distribution of selected driver alterations such as *KIT*.

One of the most notable findings of our study is the significantly higher prevalence of KIT mutations in mucosal melanoma compared to acral melanoma. While previous studies have reported KIT alterations as characteristic events in UV-independent melanomas, their reported frequencies have been variable and often inconsistent across cohorts. In this context, our results provide further evidence supporting a differential role of KIT in MM relative to AM, suggesting that KIT-driven oncogenesis may be more prominent in mucosal melanomas.

Importantly, beyond differences in mutation frequency, we also observed qualitative differences in the mutational spectrum of KIT between both subtypes. In acral melanoma, the predominance of L576P mutations is of particular interest, as this variant has been associated with sensitivity to KIT inhibitors such as imatinib. In contrast, mucosal melanoma exhibited a broader range of mutations, including variants such as T488K, Y646D, V559D, and K642E, which were not detected in acral cases. These findings raise the possibility that not only the prevalence but also the functional consequences of KIT alterations may differ between AM and MM, potentially influencing therapeutic response and disease progression.

In contrast to KIT, mutations in BRAF, NRAS, and the TERT promoter were infrequent and similarly distributed between acral and mucosal melanoma in our cohort. These results are consistent with previous reports describing a lower prevalence of canonical MAPK pathway mutations in UV-independent melanomas compared to cutaneous melanomas arising on sun-exposed skin. Together, these findings reinforce the established concept that AM and MM—as UV-independent melanomas—follow alternative oncogenic pathways, in which structural genomic alterations and non-canonical drivers may play a more prominent role than point mutations in classical melanoma genes.

From a clinical perspective, our results also highlight differences in tumour characteristics and metastatic patterns between both subtypes. Mucosal melanomas were more frequently associated with increased Breslow thickness and a higher rate of hematogenous metastasis, findings that are consistent with their well-known aggressive clinical behaviour. The higher prevalence of distant metastasis in MM could indicate potential biological differences, delayed diagnosis due to anatomical location, or a combination of both factors. These observations further suggest the possibility that AM and MM, although often grouped together, may have certain genetic differences.

The potential biological divergence between acral and mucosal melanoma may, in part, be explained by their markedly different tissue microenvironments. Acral melanomas arise in glabrous skin characterized by a thick stratum corneum and constant mechanical stress, whereas mucosal melanomas develop in non-keratinized epithelia with distinct immune and stromal contexts. These differences in epithelial architecture and local signalling environments could influence melanocyte biology, genomic instability, and selective pressures during tumour evolution. In this framework, our findings support the hypothesis that anatomical context plays a critical role in shaping the molecular landscape of melanoma.

Our study has several strengths, including the relatively large cohort for these rare melanoma subtypes and the availability of integrated clinical, histopathological, and molecular data from a single institution, ensuring consistency in diagnostic and analytical procedures. However, some limitations should be acknowledged. The retrospective design may introduce selection bias, and the number of mucosal melanoma cases remains limited due to the rarity of this subtype. Additionally, molecular analyses were restricted to a predefined set of genes and therefore do not capture the full genomic complexity of these tumours.

Future studies incorporating comprehensive genomic approaches, such as whole-exome or whole-genome sequencing, as well as transcriptomic and epigenetic profiling, will be necessary to further elucidate the biological differences between acral and mucosal melanoma, especially regarding other known melanoma drivers such as *NF1*, *CDK4*, *CDKN2A*, and other components of the *PI3K/AKT* pathway. Moreover, a deeper understanding of KIT signalling alterations and their functional consequences may help refine therapeutic strategies and identify patients who could benefit from targeted treatments.

In conclusion, our study provides insight into the hypothesis that acral and mucosal melanoma, despite sharing a UV-independent etiology, have certain differences at the clinical and biological level. The higher prevalence and distinct spectrum of KIT mutations in mucosal melanoma, together with differences in tumour thickness and metastatic patterns, encourage further research that may have implications in clinical management.

## Figures and Tables

**Table 1:** Clinical, histological, and genetic characteristics of the cohort.

	Total		AM		MM		*p* Value
	N	%	N	%	N	%	
Ulceration							
No	73/137	52.3	65/115	56.5	8/22	36.4	0.083
Yes	64/137	46.7	50/115	43.5	14/22	63.6	
Satellitosis							
No	129/135	95.6	109/113	96.5	20/22	90.9	0.253
Yes	6/135	4.4	4/113	3.5	2/22	9.1	
Breslow thickness							
≤2 mm	52/138	44.1	49/98	50.0	3/20	15.0	0.004
>2 mm	66/138	55.9	49/98	50.0	17/20	85.0	
Lymphatic metastasis							
No	81/118	68.6	69/98	70.4	12/20	60.0	0.361
Yes	37/118	31.4	29/98	29.6	8/20	40.0	
Hematogenous metastasis							
No	129/135	95.6	109/113	96.5	20/22	90.9	<0.001
Yes	6/135	4.4	4/113	3.5	2/22	9.1	
BRAF							
WT	88/101	87.1	68/80	85.0	20/21	95.2	0.292
Mutated	13/101	12.9	12/80	15.0	1/21	4.8	
NRAS							
WT	84/98	85.7	66/77	85.7	18/21	85.7	1
Mutated	14/98	14.3	11/77	14.3	3/21	14.3	
TERTp							
WT	74/78	94.9	60/62	96.8	14/16	87.5	0.185
mutated	4/78	5.1	2/62	3.2	2/16	12.5	
KIT							
WT	78/94	83.0	64/73	87.7	14/21	66.7	0.043
Mutated	16/94	17.0	9/73	12.3	7/21	33.3	
Detailed KIT mutations					K642E (1/7)		
			Dupl. 576–577 (1/9)		L576P (2/7)		
			L576P (5/9)		L642E (1/7)		
			L642E (2/9)		T488K (1/7)		
			V560D (1/9)		V559D (1/7)		
					Y646D (1/7)		

## Data Availability

The data presented in this study are available on request from the corresponding author.
